# Gender differences in physical activity motivators and context preferences: a population-based study in people in their sixties

**DOI:** 10.1186/s12889-017-4540-0

**Published:** 2017-07-04

**Authors:** Jannique G. Z. van Uffelen, Asaduzzaman Khan, Nicola W. Burton

**Affiliations:** 1 Victoria University, Institute of Sport, Exercise and Active Living (ISEAL) (office PB140), PO Box 14428, Melbourne, VIC 8001 Australia; 2 The University of Queensland, School of Human Movement and Nutrition Sciences, QLD, Brisbane, 4072 Australia; 3 Department of Kinesiology, Physical Activity, Sports and Health Research Group, KU Leuven - University of Leuven, B-3000 Leuven, Belgium; 4 The University of Queensland, School of Health and Rehabilitation Sciences, QLD, Brisbane, 4072 Australia

**Keywords:** Physical activity, Exercise, Health, Aging, Population-based study

## Abstract

**Background:**

Although regular participation in physical activity (PA) has health benefits across the life span, the proportion of people doing sufficient activity for these benefits decreases with age. The aim of this study was to identify motivating factors and context preferences for PA in people in their sixties, and to examine gender differences in these factors.

**Methods:**

Data were used from people aged 60–67 years who responded to a mail survey in Brisbane, Australia, in 2009. Respondents indicated their agreement/disagreement with seven PA motivators and 14 PA context preferences. Data were analyzed using multi-level multinomial logistic regression, adjusted for sociodemographic and health variables, and PA level.

**Results:**

Of the 1845 respondents, 59% was female. Based on self-reported PA, one in three respondents (35%) did not meet the PA guidelines of at least 150 min of moderate intensity PA per week. The three leading motivating factors for both women and men were to prevent health problems, to feel good and to lose weight. Women were more likely than men to be motivated by improving appearance (OR 2.93, 95%CI 2.07–4.15), spending time with others (1.76, 1.31–2.37), meeting friends (1.76, 1.31–2.36) or losing weight (1.74, 1.12–2.71). The three leading context preferences for both women and men were for activities close to home, at low cost and that could be done alone. Women were more likely than men to prefer activities that are with people of the same sex (OR 4.67, 95%CI 3.14–6.94), supervised (2.79, 1.94–4.02), with people the same age (2.00, 1.43–2.78) and at a fixed time (1.42, 1.06–1.91). Women were less likely than men to prefer activities that are competitive (OR 0.32, 95%CI 0.22–0.46), are vigorous (0.33, 0.24–0.47), require skill and practice (0.40, 0.29–0.55) and done outdoors (0.51, 0.30–0.86).

**Conclusion:**

Although there was overlap in motivating factors and context preferences for PA in women and men aged 60–67 years, there were also marked gender differences. These results suggest that PA options for people in their sixties should be tailored to meet gender specific interests in order to promote PA participation in this rapidly growing population group.

## Background

As a result of population ageing, the proportion of older people is increasing rapidly. Worldwide, the proportion of people aged 65+ years is predicted to increase from 8% in 2010 to 16% by 2050 [1]; this increase will be even more pronounced in developed countries. For example, the proportion of older people in Australia was 14% in 2012 and is predicted to increase to 20% by 2040 [2].

As the population ages, the number of adults with chronic health conditions and physical limitations will increase, leading to an increased burden on health care systems [1, 3]. This is particularly the case in mid-old and older-old people, as the proportion of older adults without health conditions reduces from over 30% in people in their early sixties, to just below 20% in people in their late sixties to mid-seventies and lower than 10% in people aged 75+ years [4]. Regular participation in physical activity (PA) is an important aspect of prevention and management of chronic conditions that are prevalent with increasing age, such as diabetes and cardiovascular disease [5]. Furthermore, PA has a beneficial influence on age related physical conditions such as falls and decline in functional status [6, 7], as well as on cognitive function and quality of life [7–9].

Lack of regular PA is associated with an increased risk of premature mortality. It is the fourth ranked mortality risk globally [10], estimated to cause 6–10% of deaths related to coronary heart disease, diabetes, and breast and colon cancer and 9% of premature mortality [3]. Furthermore, it contributes to increased health care costs as time spent in PA is a significant inverse predictor of subsequent numbers of prescriptions, medications, physician contacts and unplanned hospital admissions in older adults [11, 12]. Regular PA therefore is a key health behavior from a public health perspective, as it has a significant impact on health and on physical inactivity related health care costs [13]. As the numbers of older people will increase substantially over the coming decades, and age related conditions are a major contributor to the overall burden of disease [14], efforts to promote PA in this population group are critical.

In order to achieve health benefits, PA guidelines such as those from the World Health Organization [10], the American College of Sports Medicine [15] and the Australian Government [16], typically suggest that older adults do a minimum of 150 min of moderate intensity activity per week. Despite the health benefits of regular PA, the proportion of the population meeting these PA guidelines decreases with age and ranges from 30 to 60% in people aged 60+ years worldwide [17]. In Australia, only 48% of Australian adults aged 65–74 years do meet the PA guidelines and this proportion is less than 25% in adults aged 75+ years [18].

In order to encourage older adults to be sufficiently physically active, it is important to understand what motivates them to be active and how they prefer to do physical activity. Much of the evidence on successful PA programs for older people is gathered from participants in those programs and there is a need to examine PA motivators and context preferences in more representative samples of older adults [19]. Evidence from larger samples suggests that older adults have a preference for activities that are not in formal group settings. This has for example been reported in a review paper including studies in people aged 50+ years [19], in a study examining PA preferences in people aged 50–64 years and people aged 65+ years [20] and in a study including people aged 60–78 years [21]. Furthermore, another study in adults aged 60+ years reported a preference for unstructured activities [22]. Other studies have indicated preferences for PA advice from a health professional or some instruction to support PA in people aged 50+ years [20] and in those aged 60–78 years [21]. With regards to exercise in groups, adults in their sixties and seventies have a preference against exercise groups with younger people and a preference for exercise in groups with people in the same age group [23].

Although other studies have demonstrated gender differences in, for example, perceived environmental and psychosocial determinants of PA in adults aged 16–65+ years [24, 25], few have explored gender differences in motivating factors and context preferences for PA in older adults specifically. However, there are gender differences in PA participation in older adults, with women aged 50+ years [19], those aged 60+ years [26] and those aged 65+ years [27] less likely to do sufficient PA for health benefits than men. There are also gender differences in the typical ageing process, with women more likely to live longer with more health conditions [1]. This may influence the motivating factors and context preferences for PA.

The aim of this study was to identify motivating factors and context preferences for physical activity in older people and to examine if there were gender differences. As older adults are not a homogenous group, we focused on ‘young older’ adults in their sixties.

## Methods

### Sampling and data collection

Data were used from a population-based sample of ‘younger old’ adults in the HABITAT study, which assessed physical activity and associated factors among people living in Brisbane, Australia. The HABITAT baseline survey was conducted in 2007, when participants were 40–65 years. The questions about motivating factors and context preferences were included in the 2009 HABITAT survey, when respondents were 42–67 years. For this study, we included people with a minimum age of 60 years, as people aged 60+ years are referred to as older people by the United Nations [3]. After including all people aged 60 years who participated in the study, we obtained a sample of young older adults aged 60–67 years.

For the HABITAT baseline survey in 2007, a multi-stage probability sampling design was used to select a stratified random sample of 200 Census Collector’s Districts (CCD) in Brisbane. From within each CCD, a random sample of 85 people aged 40–65 years was selected, using data from the Australian Electoral Commission. Data were collected using a mail survey. Further details on sampling and data collection have been described elsewhere [28]. The HABITAT study was approved by the Queensland University of Technology Human Research Ethics Committee. As this was a mail survey, there was no direct contact between researchers and participants. Survey return was therefore taken as informed consent. This form of consent was approved by the ethics committee. Of the 10,844 surveys sent in 2009, 7837 (74% of eligible cases) respondents returned the completed surveys with data. Of these respondents, 1937 were aged sixty year or over and did not have physical limitations to do PA (i.e. responded ‘most of the time’, ‘all of the time’ or had missing data for the question ‘In the last year, has your health restricted you from doing PA?’). Data of 1845 people aged 60–67 years who provided data on their gender were included in the analysis for this study. The variable gender was missing for 92 participants, which is about 3.7% of the analytical sample.

### Measures

Respondents rated their agreement/disagreement with seven motivating factors and 14 physical activity context preferences on a five point Likert scale (*strongly disagree, disagree, unsure* [motivating factors]/ *no preference* [context preferences]*, agree, strongly agree*). The motivating factors related to health and wellbeing (e.g. ‘to make me feel good’, ‘to reduce stress’), appearance (e.g. ‘to lose weight’), and social aspects of PA (e.g. ‘to meet new friends’). Questions about the PA context preferences have been used in previously published research and assessed format (7 items), location (2 items) and social setting (5 items) [29–31]. The original selection of these study variables in the HABITAT study was guided by social cognitive theory which purports that behavior is influenced by personal, environmental, and behavioral factors [32]. When applied to understanding physical activity behaviour [33], personal factors include demographic and biological variables (e.g., gender, age, weight, health); environmental factors include social contextual variables (e.g., supervision, competition, meeting people) and physical contextual variables (e.g., location, cost); and behavioral factors reflect attributes of physical activity (e.g., intensity, skill required, format). Details about questions are provided in Tables 2 and 3 and complete questions are included in Appendices A-B. Responses were skewed towards extreme values (i.e. strongly agree, strongly disagree) and were therefore collapsed into three categories (*disagree*, *unsure* [motivating factors]/ *no preference* [context preferences], *agree*).

Sociodemographic characteristics included gender, age, education, employment status, income and living arrangements. These were assessed using standard questions and categorized as detailed in Table 1.

**Table 1:** Sociodemographic characteristics, health-related characteristics and physical activity level of the respondents stratified by gender (*n* = 1845)

	Female (*n* = 1089)		Male (*n* = 756)		*P*-value
*Sociodemographic characteristics*					
Age (Mean [SD])	63.3	2.17	63.0	2.25	<.05
	n	(%)^a^	n	(%)^a^	
Highest Educational Qualification					**.001**
School only (up to 12 years)	582	(54)	272	(36)	
Certificate/Diploma	247	(23)	265	(35)	
University degree	252	(23)	219	(29)	
Employment Status					**.001**
Full time paid	196	(20)	308	(44)	
Part time/Casual paid	212	(22)	116	(16)	
Not in paid work	164	(17)	36	(5)	
Retired	403	(41)	246	(35)	
Gross Annual Household Income (AUD)					**.001**
<41,600	419	(49)	223	(32)	
41,600–72,799	237	(28)	217	(31)	
72,800–129,999	135	(16)	163	(24)	
≥130,000	69	(8)	88	(13)	
Living arrangements					**.01**
Living alone	287	(30)	176	(25)	
Living with partner	572	(59)	397	(57)	
Living with partner and children	109	(11)	121	(17)	
*Health related characteristics and physical activity*	n	(%)^a^	N	(%)^a^	
Self-rated health					.53
Excellent/Very good	490	(45)	311	(42)	
Good	468	(43)	328	(45)	
Fair/Poor	131	(12)	94	(13)	
Body Mass Index (kg/m^2^)					**<.001**
<25	453	(42)	234	(31)	
≥25–29.9	399	(37)	363	(48)	
≥30	217	(20)	159	(21)	
Psychological Distress					.87
No distress	989	(93)	695	(93)	
Distress	69	(7)	50	(7)	
Physical activity level					.21
None	134	(13)	104	(14)	
Some	231	(22)	137	(19)	
Meeting guidelines^b^	672	(65)	481	(67)	

*AUD* Australian dollar, *n* number, *SD* standard deviation; ^a^Column percentages may not add up to 100 because of rounding; ^b^Equivalent of at least 150 min/week of moderate intensity physical activity. Boldface indicates statistical significance (*p* < .05)

Health-related variables included general health, body mass index (BMI), and psychological distress. Respondents were asked to rate their health as *poor, fair, good, very good* or *excellent*. BMI was based on self-reported height and weight and categorized according to the World Health Organization classification (<25 kg/m^2^, 25- < 30 kg/m^2^ or >30 kg/m^2^) [34]. Psychological distress was assessed using the Kessler 6 scale, a reliable and validated scale to screen for mental health issues in population based surveys [35]. Respondents indicated the frequency of feeling sad, nervous, restless, hopeless, worthless, and that everything was an effort in the past week on a five point Likert scale ranging from *none of the time* [0 points] to *all of the time* [4 points]. The responses were summed and categorized into no distress (0–7 points) or some distress (8–24 points), using cut off points as identified previously [36].

Physical activity was assessed with the items from the Active Australia questionnaire [37], which has been used frequently in population based surveys and has acceptable reliability and validity in mid aged and older adults [38, 39]. Respondents reported time spent walking briskly (3.33 Metabolic equivalents [METs]), in moderate (3.33 METs) and in vigorous leisure time PA in the previous week (6.66 METs). The sum of the products of time in each of these activities and the MET-value was calculated and categorized as no PA (<90 MET.minutes), some PA (90–449 MET.minutes), or meeting PA guidelines (≥450 MET.minutes). The category ‘meeting guidelines’ is comparable with PA guidelines of at least 150 min of moderate-intensity PA per week [10, 15, 16].

### Statistical analysis

Data were analyzed in Stata using multilevel multinomial logistic regression to reflect the multistage sample selection. A random intercept for CCD was included in the regression models to account for clustering within CCDs. The regression analysis with random effects was conducted using GLLAMM (generalized linear latent and mixed models) commands of STATA version 11.0 (StataCorp, College Station, TX, USA). The assumption of parallel regression, essential for ordinal logistic regression, was assessed using the Brant test and the estimation procedure used was numerical integration (10 integration points) with adaptive quadrature in order to obtain more reliable estimates of parameters [40]. The multinomial logistic regression analysis used to examine the association between gender (i.e. independent variable) and preference for certain motivating factors and context preferences (i.e. dependent variable) was adjusted for possible confounding effects of sociodemographic characteristics (age, education, employment status, income, living arrangements), health-related variables (self-rated health, body mass index, psychological distress) and physical activity level. A significance level of 0.05 was used to indicate statistical significance and odds ratios and 95% confidence intervals are reported. The odds ratio’s represent the likelihood of women agreeing with each of the motivating factors or context preferences (versus ‘disagreement’), compared to men.

## Results

### Participants

Mean age of the 1845 respondents was 63.2 (SD 2.21) years and 59% was female. Female respondents were slightly older than the male respondents (*p* < .05, see Table 1). There were some gender differences in sociodemographic characteristics, with more men than women having a higher education, fewer men not in paid work and more in a full time paid position, and men having higher income. Men were also less likely to live alone and more likely to live with their partner and children (all *p* < .01, see Table 1). Most people rated their health as good-excellent and a higher proportion of men had a BMI >25 kg/m^2^ (*p* < .05, see Table 1). There were no gender differences in physical activity levels; 65% of respondents met PA guidelines, 21% did some PA and 14% did no PA.

### Motivating factors

The proportion of women and men indicating agreement or disagreement with each of the seven motivating factors is presented in Table 2. The leading three motivating factors for PA for both women and men, were ‘to prevent health problems’ (97% of women and 96% of men), ‘to feel good’ (95% of women and 91% of men) and to ‘lose/manage weight’ (88% of women and 84% of men). The social factors ‘spend time with others’ (68% of women and 52% of men) and ‘meet new friends’ (58% of women and 38% of men) were endorsed by fewer respondents than the health and wellbeing-related factors. There were significant gender differences in the likelihood of endorsing several factors (Table 2). After adjusting for age, education, employment status, income, living arrangements, self-rated health, body mass index, psychological distress and physical activity level, women were more likely than men to agree with motivators of improving appearance (OR 2.93, 95%CI 2.07–4.15), spending time with others (OR 1.76, 95%CI 1.31–2.37), meeting friends (OR 1.76, 95%CI 1.31–2.36) and losing weight (OR 1.74, 95%CI 1.12–2.71).

**Table 2:** Agreement with motivating factors stratified by gender and gender differences in the likelihood to agree

	Agree (%)^a^	Disagree (%)^a^	OR 95%CI for women compared to men^b^
Prevent health problems			
Women	97	1	2.11 (.76–5.82)
Men	96	3	1.00
Make me feel good			
Women	95	3	1.42 (.71–2.84)
Men	91	6	1.00
Lose or manage weight			
Women	88	9	**1.74 (1.12–2.71)**
Men	84	11	1.00
Help manage stress			
Women	85	8	1.52 (.97–2.37)
Men	80	10	1.00
Improve appearance			
Women	81	13	**2.93 (2.07–4.15)**
Men	60	24	1.00
Spend time with others			
Women	68	22	**1.76 (1.31–2.37)**
Men	52	29	1.00
Meet new friends			
Women	58	27	**1.76 (1.31–2.36)**
Men	38	36	1.00

OR 95%CI: Odds Ratio 95% Confidence Interval

Boldface indicates statistical significance (*p* < .05)

^a^Row percentages do not add up to 100%, as percentages for ‘Unsure’ are not reported

^b^Results from multilevel multinomial logistic regression: Odds ratio for ‘agreement’ versus ‘disagreement’ (not taking people who responded ‘unsure’ into account), adjusted for age, education, employment status, income, living arrangements, self-rated health, body mass index, psychological distress and physical activity level

### Physical activity context preferences

The proportion of women and men indicating agreement or disagreement with the PA contexts relating to format, location and social setting is reported in Table 3. The leading three PA context preferences, endorsed by more than two-thirds of the respondents, were ‘activities close to home’ (87% of women and 78% of men), at ‘little or no cost’ (80% of women, 73% of men) and activities that ‘I can do on my own’ (68% of women, 77% of men). For activity format, women were more likely than men to prefer activities at a fixed time (OR 1.42, 95% CI 1.06–1.91) and less likely to prefer activities that are competitive (OR 0.32, 95%CI 0.22–0.46), vigorous (OR 0.33, 95%CI 0.24–0.47), skilled (OR 0.40, 95%CI 0.29–0.55), or done outdoors (OR 0.51, 95%CI 0.30–0.86). In terms of social setting, women were more likely to prefer activities with people of the same sex (OR 4.67, 95%CI 3.14–6.94), supervised activities (OR 2.79, 95%CI 1.94–4.02), and activities with people of the same age (OR 2.00, 95%CI 1.43–2.78). Women tended to be less likely than men to prefer activities that could be done alone (OR 0.64, 95%CI 0.40–1.00).

**Table 3:** Agreement with context preferences stratified by gender and gender differences in the likelihood to agree

	Agree (%)^a^	Disagree (%)^a^	OR 95%CI for women compared to men^b^
*Format*			
Little or no cost			
Women	80	8	1.30 (.81–2.08)
Men	73	9	1.00
Are not just about exercise			
Women	63	14	1.13 (.77–1.68)
Men	55	15	1.00
Have a set routine or format			
Women	52	23	.79 (.56–1.10)
Men	50	19	1.00
Done at a fixed time			
Women	44	35	**1.42 (1.06–1.91)**
Men	32	41	1.00
Require skill and practice			
Women	23	47	**.40 (.29–.55)**
Men	34	1	1.00
Are vigorous			
Women	24	51	**.33 (.24–.47)**
Men	36	30	1.00
Involve competition			
Women	12	73	.**32 (.22–.46)**
Men	22	52	1.00
*Location*			
Close to home			
Women	87	4	1.45 (.78–2.68)
Men	78	5	1.00
Done outdoors			
Women	62	10	**.51 (.30–.86)**
Men	72	5	1.00
*Social setting*			
Can do on my own			
Women	68	12	.64 (.41–1.00)
Men	77	8	1.00
Done with people my age			
Women	58	18	**2.00 (1.43–2.78)**
Men	42	25	1.00
Done with people my own sex			
Women	33	31	**4.67 (3.14–6.94)**
Men	11	43	1.00
Involve supervision			
Women	26	48	**2.79 (1.94–4.02)**
Men	12	63	1.00
Are team-based			
Women	11	63	.71 (.46–1.09)
Men	12	60	1.00

*OR 95%CI* Odds Ratio 95% Confidence Interval

Boldface indicates statistical significance (*p* < .05)

^a^ Row percentages do not add up to 100%, as percentages for ‘No preference’ are not reported

^b^ Results from multilevel multinomial logistic regression: Odds ratio for ‘agreement’ versus ‘disagreement’(not taking people who responded ‘no preference’ into account), adjusted for age, education, employment status, income, living arrangements, self-rated health, body mass index, psychological distress and physical activity level

## Discussion

Regular physical activity (PA) has health benefits across the life span, and among older adults there are specific benefits for physical, psychological and cognitive wellbeing. However, the proportion of people being sufficiently active for health benefits decreases with age. To promote PA in an ageing population, it is critical to have knowledge about what motivates older people to be active and what their preferences are, so as to optimize potential appeal and engagement. This study identified motivating factors and context preferences for PA in community dwelling adults aged 60–67 years and examined gender differences.

### Motivating factors

People in this age group were more likely to be motivated by factors related to health and wellbeing, than by social factors. Almost all men and women endorsed preventing health problems and feeling good as motivating factors to do physical activity. This is in line with the findings of several systematic reviews summarizing PA motivators in people aged 50+ years [41], 65+ years [42], and 80+ years [43].

Healthier people are more likely to participate in and adhere to exercise programs for preventative benefits [42]. However, half of the people aged 65+ years have multiple long term health conditions or disabilities [44]. It may therefore be important to not only promote the preventative benefits of PA, but to also promote the potential benefits of regular PA for those with established health conditions, as research has demonstrated that PA can improve health related quality of life in older adults with physical [45, 46] and psychological conditions [47, 48]. This is especially important as health problems are a key barrier to engage in PA for people aged 65+ years [42] and those aged 80+ years [43]. The potential of a vicious cycle, in which people become less active because of their health problems, which in turn could result in further deterioration of their health, should be avoided. This could be done by ensuring that PA options and programs can be adapted to cater for the abilities of people with health problems [49]. The provision of these programs becomes more relevant as people age and the prevalence of chronic conditions and health problems affecting mobility and motivation increases. Although this may not be needed yet for people in their sixties, it is certainly relevant to think about potential adaptations to programs to ensure people in their seventies and eighties and ideally nineties and further can continue participation in PA programs. In addition, tailored programs could be developed for people with specific conditions, as has for example been done for cancer and arthritis [50, 51], to cater for the specific issues these people may face and provide a supportive environment for PA.

The motivating factor to “feel good” could also be used to promote PA opportunities for young older adults, including those with established health conditions. This may relate to enhancing positive wellbeing and self-image; as well as managing psychological difficulties. There is a positive association between PA and positive perceptions of ageing in people aged 65–85 years [52], between PA and happiness in people in their seventies [53], and between PA and subjective wellbeing in people aged 60–64 years, even after accounting for long term illness [54]. Even low levels of PA can reduce the risk of future depression and anxiety in women in their seventies [55], and among those 50+ years [56] and those in their fifties and sixties [47] with depression, PA can improve mood, and physical and psychological health-related quality of life.

Social factors, such as spending time with others and meeting new friends, were endorsed by lower proportions of young older men and women than health related factors. This seems to contradict previous research identifying social support as a key motivator for PA in people aged 50+ years [41] and in those aged 80+ years [43]. It may be, however, that health and wellbeing benefits are valued more highly by young older adults when considering PA adoption, and that social factors are important for maintaining PA participation. Other research has identified a positive association between social support and adherence to programs and maintenance of physical activity [49, 57]. Social support is a key determinant of healthy ageing [58], and PA programs may provide an excellent opportunity to create social networks to provide peer-support [49].

There were marked gender differences among young older adults in motivating factors related to appearance, weight and social factors. Women were two to three times more likely than men to be motivated by losing or managing weight and improving appearance. This is in line with findings for young girls and women [41]. The association between weight and health is complex in women as they age [59, 60]. Intentional weight loss may benefit health and decrease the risk of chronic conditions in overweight and obese women in their fifties and seventies [60], however, there is also evidence that weight loss in adults in their late forties to mid-seventies can be a risk factor for age related conditions such as osteoporotic fractures [61]. Our finding that women in their sixties were almost twice as likely as men in their sixties to be motivated by social factors confirms previous findings in women aged 50+ years [19] and gender differences in social influence for exercise adoption [62].

### Context preferences

Context preferences for PA have been less frequently examined than motivating factors. This study showed that more than three out of four young older adults preferred activities at little or no cost, close to home, and that they could do alone (men only). The preference for low cost activities reflects previous research where costs were reported as a main barrier to PA by people aged 50+ years [41] and those aged 80+ years [43]. The preference for activities close to home is in line with previous findings that programs at inconvenient locations are associated with decreased participation by adults aged 50+ years [19], and that adults aged 45+ years are less inclined to travel longer distances to PA opportunities than younger adults [63]. From a public health perspective, it is important therefore, that all young older people have access to PA options and programs, and low cost activities that can be done in their local area. Proximal destinations, and destinations that also facilitate some social interaction, have been identified as a key predictor of walking in adults aged 65+ years [64, 65] and highlight the importance of a neighbourhood environment that is conducive to walking. Other environmental factors that are associated with more PA in this age group include safety, pedestrian infrastructure and connectivity, access to retail and services and aesthetics [66, 67]. Our Findings that adults in their sixties preferred activities that could be done alone is consistent with other research that a large proportion of people aged 50+ [19] and 60+ years [22] have a preference against structured classes. This may reflect concerns with keeping pace with younger people of higher ability, or being held back by older people of less ability.

There were marked gender differences among the young older adults in preferences for PA format, location and social setting. Women preferred activities at a fixed time, whereas men were more likely to prefer activities that require skill and practice, that are vigorous and that involve competition. In terms of location, men were more likely to prefer outdoor activities. In terms of social setting, women were more likely to prefer activities with people the same age, supervised activities and activities with other women. Thus, women have stronger preferences than men for who they are active with, which matches with the findings that they are more likely to be motivated by social factors [19]. Men had stronger preferences for the style of PA; their preference for skilled, vigorous, competitive and outdoor PA is characteristic of sports. Sports based PA options could therefore be appealing for young older men. There is a perception among men however, that the majority of community based sporting opportunities are primarily for young people [68]. As sport participation is associated with psychological and social health benefits [69] and because of the clear role of sports based PA for public health [70], the option to promote PA participation in young older men through sports based PA options should be further explored.

### Study strengths and limitations

This study fills a gap in the literature by providing knowledge about PA motivators and context preferences in a population based sample of young old adults. This new evidence can inform the development of PA interventions for people in their sixties, who can have different interests from mid aged adults [30]. In contrast to many previous studies, these data were from a large population based sample, rather than from people participating in PA programs [19]. The results are therefore, more generalizable and less likely to reflect individual experiences with specific programs. Because of the large sample, it was possible to adjust the analyses for a range of sociodemographic and health related factors and PA. This study focused on adults in their sixties and so results may not be generalizable to adults older than this. In addition, respondents were from one major city in Australia, which may limit generalizability to those in other major cities or more regional areas. The style of assessment may have influenced results. For example, “preventing health problems” was provided as one possible motivator, but “managing current health problems” was not. It may be, therefore, that respondents who endorsed preventing health problems were motivated by potential health benefits but not prevention per se. Finally, contextual preferences may not always reflect the actual physical activity participation, which may be influenced by other factors such as available opportunities and logistics.

### Implications and conclusion

PA opportunities for adults in their sixties should emphasize “preventing health problems” and “feeling good” as benefits as these were the main motivating factors for both men and women. Although women in this age group were also motivated by weight loss/management, caution may be needed given the potential health risks of low BMI among older adults. It may therefore be more useful to highlight benefits related to improved appearance and social opportunities, which were also important to women aged 60–67 years.

In terms of PA context preferences of adults in their sixties, it is important to prioritize PA options that are low cost, that can be done close to home and options outside of groups, as these were the key preferences. In addition, there were marked gender differences and there is a need to take these into account in the promotion and development of PA options for this age group. PA opportunities for women in their sixties may be more appealing if they are for women only, older adults only, supervised, and done at a fixed time. Men in their sixties may be more attracted to PA opportunities that have a degree of competition, are vigorous, skill-based, and outdoors.

Thus, although there is overlap in motivating factors and context preferences for PA in adults in their sixties, there are also marked gender differences. The results of this study suggest that PA options for people in their sixties should be tailored to meet gender specific interests in order to promote regular PA participation in this rapidly growing population group.

## Appendix 1

List of items used to assess motivating factors.

There are different reasons why people might do physical activity. Which of these reasons could motivate you to do physical activity?

1. To prevent health problems
2. To help manage stress
3. To lose weight, or manage my weight
4. To spend time with others (e.g. friends, family, partner)
5. To improve my appearance
6. To make me feel good
7. To meet new friends

Response options: Strongly disagree; Disagree; Unsure; Agree; Strongly agree.

## Appendix 2

List of items used to assess preferred activity context.

If you had the choice, what sort of physical activities would you prefer to do?

I would prefer to do activities that:

1. I can do on my own
2. Involve competition
3. Are done with people around my own age
4. Can be done close to home
5. Are done outdoors
6. Require skill and practice
7. Have a set routine or format
8. Involve little or no cost
9. Involve supervision (e.g., from a leader)
10. Are team based
11. Are done at a fixed time (i.e., scheduled sessions)
12. Are done with people of my own sex
13. Are not just about exercise
14. Are vigorous

Response options: Strongly disagree; Disagree; No preferences; Agree; Strongly agree.

## Abbreviations

95% CI: 95% confidence interval
AUD: Australian dollar
BMI: Body mass index
CCD: Census collector’s district
GLLAMM: Generalized linear latent and mixed models
MET: Metabolic equivalent
n: Number
OR: Odds ratio
PA: Physical activity
SD: Standard deviation
